# Effect of supersaturation on hillock of directional Growth of KDP crystals

**DOI:** 10.1038/srep06886

**Published:** 2014-11-03

**Authors:** Fa-Fu Liu, Guang-Wei Yu, Li-Song Zhang, Liang Li, Bo Wang, Xiao-Yu Gan, Hong-Kai Ren, Hai-Liang Zhou, Li-Li Zhu, Shao-Hua Ji, Ming-Xia Xu, Bao-An Liu, Xin-Guang Xu, Qing-Tian Gu, Xun Sun

**Affiliations:** 1 State Key Laboratory of Crystal Materials, Shandong University Jinan 250100, China; 2 School of Information Science and Engineering, Shandong Agricultural University Taian 271018, China; 3 Advanced Research Center for Optics, Shandong University Jinan 250100, China; 4 Key Laboratory of Functional Crystal Materials and Device, Ministry of Education, Shandong Universiy Jian 250100, China

## Abstract

KDP single crystals were grown in aqueous solution by using “point seeds” with a defined crystallographic direction of 59° to the Z axis. When hillock slopes on the (100) face of KDP crystals were measured within the supersaturation (*σ*) range of 0 < *σ* ≤ 0.06, the slope of hillocks with hollow cores depended nonlinearly on supersaturation. Below *σ* = 0.02, the hillock slope depended on supersaturation, but when *σ* was ≥ 0.02, the hillock slope increased more gradually and was less dependent on supersaturation. Hollow funnel-shaped growth dislocation on the (100) face of KDP crystals was observed at *σ* = 0.04, characterized by large holes with micro-steps and step bunching inside, the formation of which were analyzed. The result verified that the reversed growth appears to occur within hollow channels found on growth hillocks.

Potassium Dihydrogen Phosphate (KDP) single crystals are very useful materials for many applications, such as production of frequency multipliers of laser radiation^1^,^2^,^3^. Large size KDP single crystal growth using “z plate” or “point seed” methodology has been well characterized. Also, the growth characteristics and the effect of supersaturation have been reported^4^,^5^,^6^,^7^. KDP crystals grown at *σ* < 0.25 were generated largely by a screw dislocation mechanism, which was verified using atomic force microscopy (AFM) scanning^2^. A study of the evolution with time of step roughness on KDP crystal faces with high densities of kinks has also been done using AFM^3^. Mariusz *et al.*^4^ used in situ AFM to study the surface morphology of the (100) face of a KDP crystal, and observed step pinning. Two dislocation spirals of different signs on the prism face of the KDP crystal were observed using AFM in 1999^5^. In 2004, more detailed works investigating the morphology of the (100) face were reported by Thomas *et al*^6^. Using AFM, they observed growth dislocation sources with various Burgers vectors on the (100) face of KDP crystals. However, they did not observe growth hillocks with hollow cores on the (100) face. They observed that the hillock slope linearly depended on both supersaturation and hillock geometry. In contrast, De Yoreo *et al.*^7^ observed hillocks with hollow cores on the (101) face of KDP crystals in the supersaturation range of 0.03 ≤ *σ* ≤ 0.31, where hillock slope depended nonlinearly on supersaturation. The above investigations were all based on KDP single crystals grown using Z-cut point seeds and were analyzed using AFM. However, the study of the surface morphology of a single KDP crystal grown in a defined crystallographic direction of 59° has not yet been described.

In this work, KDP crystals were grown in an aqueous solution using point seeds with a defined crystallographic direction of 59° to the Z axis (see Figure 1 a, b). In comparison with the crystal grown using a conventional Z-cut seed, the prism faces of the KDP crystal grown in our experiment are not parallel to the vertical direction of the crystallizer (see Figure 1c). Hillock slopes on the (100) face of KDP were measured within the supersaturation range of 0 < *σ* ≤ 0.06. We found that the slope of hillocks with hollow cores increased nonlinearly with supersaturation values ≤ 0.02, but for *σ* ≥ 0.02, hillock slopes increased more gradually and leveled off. A model was established to explain this phenomenon. Using this model, mathematical relationships were developed to predict the correlation between hillock slope and supersaturation. This model agrees well with our observations on the crystal growth on the (100) face of KDP.

## Results

### Structure of growth hillocks

Previous research has shown that the growth rate of the surface of a crystal is closely related to the hillocks on its surface. Land and De Yoreo^8^ demonstrated that under certain conditions, dislocation sources form due to the incorporation of micro-crystals, which subsequently act as growth sources by stacking onto the hillocks. In our experiments, when the supersaturation level reached 0.02, hillocks with hollow cores on the (100) face of KDP were observed. As shown in Figure 2, the hillocks possessed two-fold rotational symmetry, corresponding to the space group (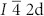) of the KDP crystal. The hillocks were oriented along the four step directions on the (100) face and the rounded sections at the top of hillock, apparent in ex situ AFM morphology results, resulted from post-growth annealing^9^. Depending on the ex situ image, incorporation of steps emerging in the hollow cores were verified.

Figure 3 and Table 1 show the dependence of hillock slope on supersaturation values for *σ* between 0 and 0.06. The data shows that the hillock slope rises abruptly until *σ* = 0.02, beyond which it increases more slowly and levels off.

### Hollow dislocation-growth source

As shown in Figure 4, a special spiral dislocation on the (100) face of the KDP crystal grown at *σ* = 0.04 was observed with AFM. Its morphology, which appeared to be a funnel-shaped hole, does not resemble hillocks reported previously^6^. The hollow core was a rounded parallelogram, which exhibited two-fold rotational symmetry, namely the symmetry in the crystallization direction of [100] or [010], given that the space group of KDP crystal is 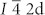. The depth of this hole was about 420 nm, and contained numerous micro-steps with heights ranging from 6.3 to 50 nm, which grew and were incorporated inside it. This reverse crystal growth occurred on the (100) face of the KDP crystal.

### Formation of the hillocks with hollow core

Classical nucleation theory allows us to know that a critically spontaneous nucleation radius (r*_c_*) decreases with increasing supersaturation: 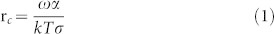
where *ω* is the inverse of the number density of molecules in the solid, α is the free energy of the step edge per unit step length per unit step height, and the value of them shows in Table 2, *k* is the Boltzmann constant, *T* is the Kelvin temperature of crystal growth, and *σ* is the supersaturation value. As shown in Figure 5, the breadth of the meta-stable region becomes narrow with decreasing growth temperature. As previously reported, if the supersaturation of the KDP growth solution was large enough to exceed demand for solute by the meta-stable boundaries of growth, micro-crystals will appear in solution^9^. These micro-crystals will land on either the (100) or (101) face of the KDP crystal and begin their growth. De Yoreo *et al.*^11^ postulated that particles floating in solution are more likely to land on the upward-facing surfaces (pyramidal faces) because of their proximity to that surface, resulting in the bias toward inclusion formation in the pyramidal sectors of the crystals.

In our experiments, the (100) face of KDP crystals grown with point seeds, whose crystallographic direction is 59° with respect to the Z axis, are upward-facing and more available to bind to most of the micro-crystals floating randomly in solution^7^,^11^. Once a particle randomly lands on a (100) face, an inhomogeneous surface supersaturation field is created due to the difference of the velocity of solute diffusion to the upward-facing (100) face, and subsequent macro-steps propagate the instability, resulting in lattice defects^13^. Each defect would then become an origin of a dislocation with a large strain field^10^,^11^. Several research teams have postulated that the resulting strain field associated with a dislocation would produce a hollow core^14^,^15^,^16^. In short, crystal growth under these conditions will produce hillocks with hollow cores on crystal faces that are not parallel to the vertical direction of the crystallizer.

### The effect of a hollow core on the slope of hillocks

Burton, Cabrera, and Frank^17^ proposed the classically basic relationship between the structure of a dislocation source and the vicinality of the resultant growth hillock during the growth of single crystals. Within this sample model (BCF model), hillock slope value can be derived when the supersaturation, the temperature, the free energy of the step edge and the height of an elementary step are available; the hillock slope, P, for an isotropic screw dislocation can be derived by^17^

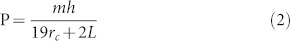
where m is the number of unit steps in Burgers vector, *h* is the height of a single step, whose value shows in the Table 2, and 2*L* is the length of the perimeter at the surface surrounding the group of dislocations which create the hillock. When *L* = 0, P is proportional to supersaturation. However, *L* is not equal to zero in reality, so the dependence of hillock slope on supersaturation predicted by the BCF model does not correlate linearly with supersaturation^7^.

When a dislocation source on the (100) face generates a hollow core on the top of a hillock, the steps must spiral around the hollow core. Thus, we expect that 2*L* is equal to 2πr_0_ rather than zero. A model of a hillock with a hollow core is shown in Figure 6; *L_1_* and *L_2_* are roughly equal to the lengths of the chords of the hollow core. A parallelogram spiral makes one full rotation about a core of radius r_0_. Because of its two-fold rotational symmetry, the model can be described by the following equations: 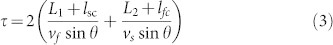

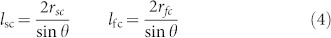

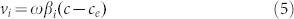

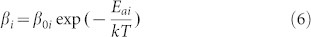
where time of one full rotation about a core of radius r_0_ is given by 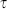, *θ* is the acute angle of hollow parallelogram core, *v_s_* and *v_f_* are the step movement velocity of the slow side and fast side respectively, r*_sc_* and r*_fc_*are the critical radii of the slow and fast sides, respectively, *l_sc_* and *l_fc_* are the critical length of the slow and fast sides, respectively, *β_i_* is a kinetic factor, *E_ai_* is energy barrier, the value of *β_0i_* and *E_ai_* shows in Table 2, and *c_e_* and *c* are the equilibrium and actual concentrations of KDP salt, respectively. The hillock slope of the *i*th sector is given by 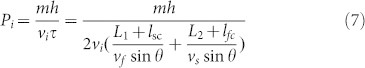


The hillock slope versus supersaturation along with curves predicted by equation (7) is shown in Figure 7, which agrees well with the experimental data when m is equal to 10 at a supersaturation range between 0 and 0.06. The *l_sc_* or *l_fc_* decreased while supersaturation increased, *l_sc_* and *l_fc_* are about 20 nm when *σ* = 0.06, and the value of them will decrease largely with increases in supersaturation. However *L*_1_ and *L*_2_ are on the order of hundred nanometers and the values of *L*_1_ and *L*_2_ are much larger than *l_sc_* and *l_fc_*, thus *L*_1_ + *l_sc_* ≈ *L*_1_, *L*_2_ + *l_fc_* ≈ *L*_2_, equation (7) can be changed to: 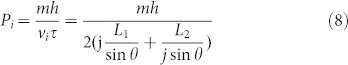

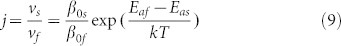


From equations (8) and (9), the hillock slope, P, becomes constant gradually with the increase in supersaturation, as verified by the experimental data shown in Figure 3.

De Yoreo *et al.*^7^ analyzed the effect of supersaturation on the slope of growth hillocks with holes on the (101) face of a KDP crystal and found that the slope of hillocks with a hollow core tend to be independent of supersaturation. From our experiments, we have also reached a similar conclusion, that the slope of hillocks with hollow core slowly reach a limiting value when *σ* ≥ 0.02; the slope of hillocks with a hollow core on the (100) face correlates with the size of the hollow core rather than the supersaturation value.

### The analysis of the formation of reversed growth

Hollow dislocation-growth in our experiment can be regarded as a kind of reversed crystal growth. As we know, reversed crystal growth in nanocrystals has been described in detail^18^,^19^, which basically follows a sequence of steps: 1) amorphous aggregates get together to form a disordered cluster; 2) the surface of the disordered cluster crystallizes first; 3) finally, the interior of cluster crystallizes from surface to core. The growth of the (100) face of KDP crystals had been investigated in previous work^4^,^20^,^21^,^22^, but no such reversed growth had been reported. However, in our growth study, when supersaturation is 0.04, the reversed growth occurred on the (100) face of the KDP crystal. From the experimental data and conclusion above, we deduce that the formation of the reversed growth in a single crystal occurs if the following conditions are met: 1) there is a big funnel-shaped hole on growth face which favors entry of growth-supplying solutes and 2) the supersaturation of the growth solution is moderate.

## Discussion

Using AFM scanning, hollow cores have been found in hillocks on the (100) face of KDP crystals which were grown using point seeds with a defined crystallographic direction of 59° with respect to the Z axis. With increasing supersaturation, the hillock slopes rise slowly and approach a constant when *σ* is ≥ 0.02; above this value, the slopes depend on the geometry and size of hillocks with holes, rather than on supersaturation. Taken together, the conclusion reached by other groups^11^,^12^ when pooled with our observations, indicate that hillocks with hollow cores form easily on the faces that are not parallel to the vertical direction of crystallizer. Reversed crystal growth in large holes occurs readily, demonstrating that the solution in the holes not only forms inclusions, but also supplies growth units. The present results reveal a new phenomenon of single crystal growth, which warrants further investigation.

## Methods

### Material preparation

**Figure 1:**
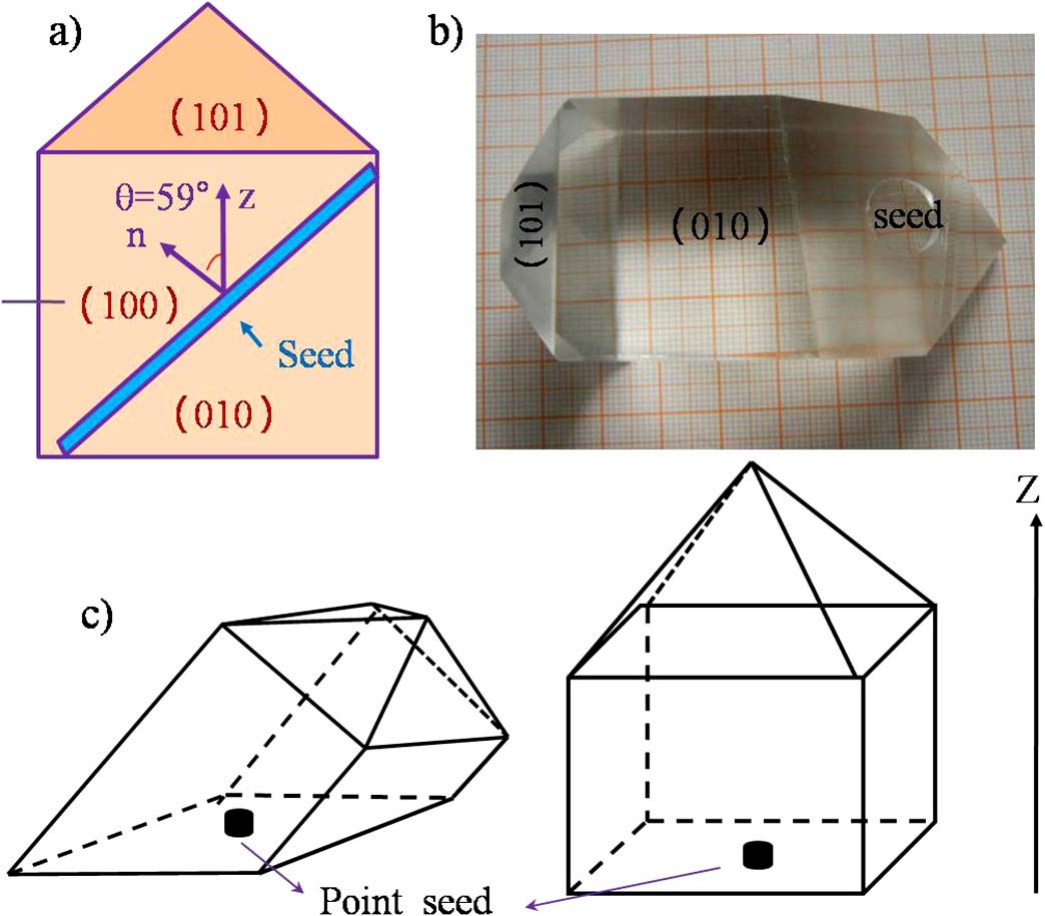
General view of the seed, (a), and a crystal grown from seed in defined direction of 59° with respect to Z axis, (b), the schematic diagram of KDP single crystals grown with seeds of 59° to Z axis (the left) and Z-cut seeds (the right), (c). (The black arrow represents the vertical direction of crystallizer).

**Figure 2:**
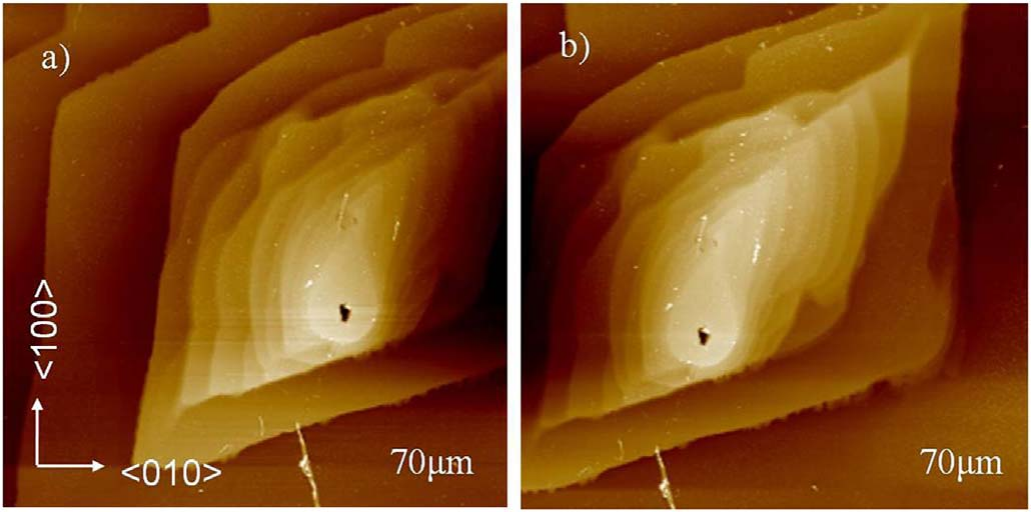
Ex situ AFM image of growth hillock of (100) face of KDP crystal grown at *σ* = 0.02, (a) and (b) represent left and right section of which, respectively.

**Figure 3:**
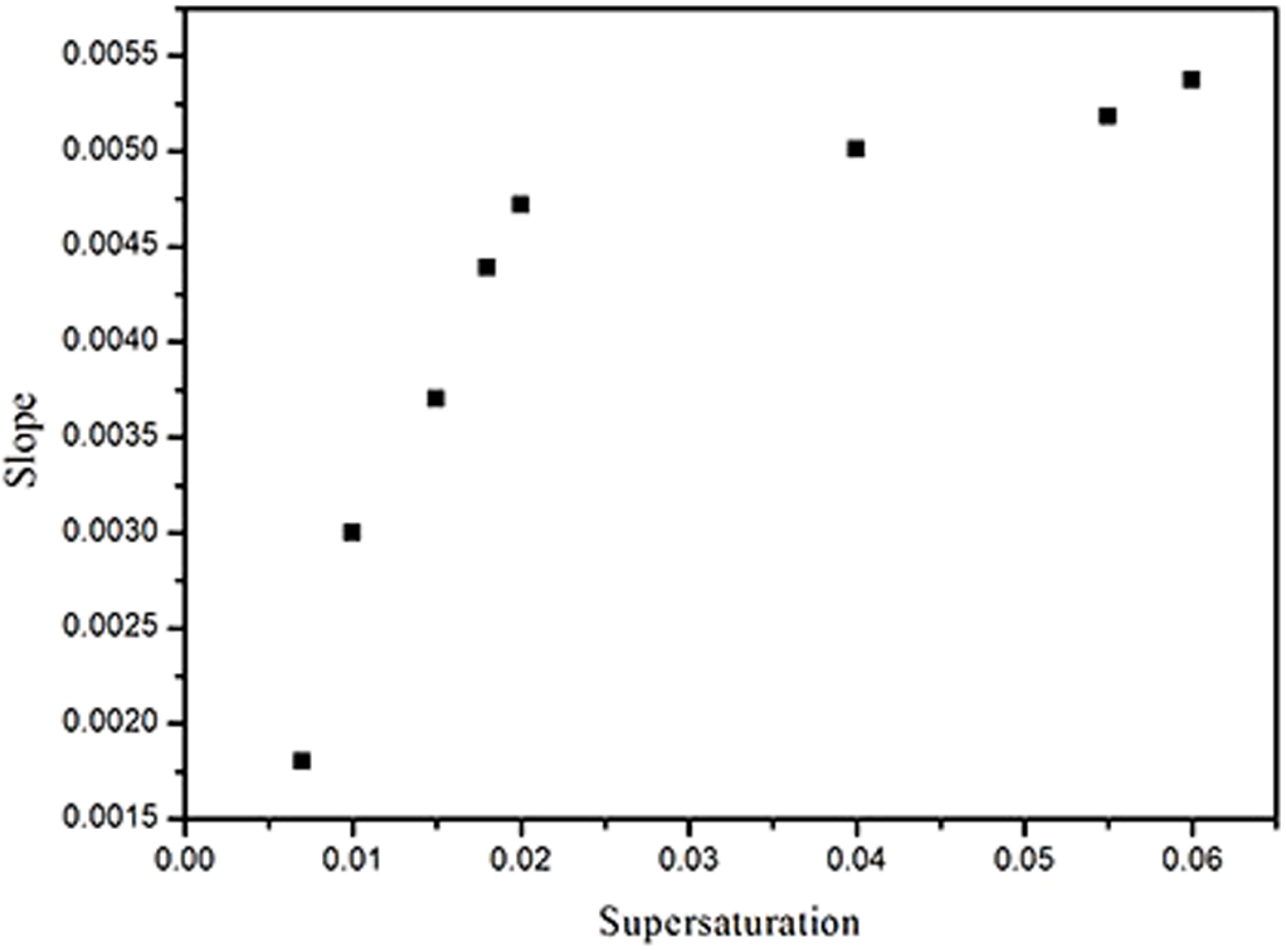
Average value of hillock slope vs. supersaturation.

**Figure 4:**
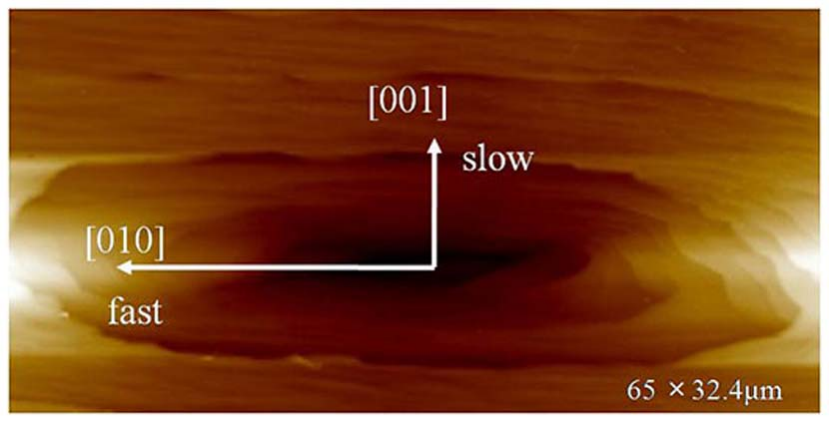
AFM topology of a growth dislocation hole on (100) face of KDP grown at *σ* = 0.04, (65 × 32.4 μm).

**Figure 5:**
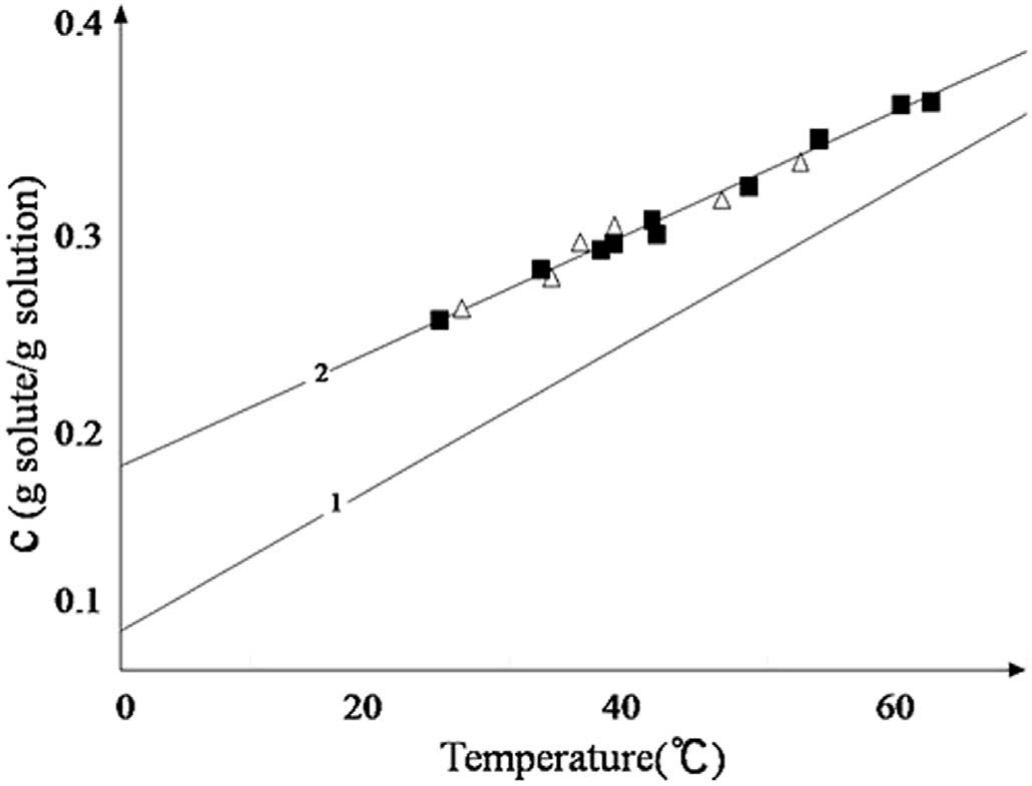
Stability of supersaturated KDP solutions: (1) solubility curve (2) meta-stable boundaries of solutions with (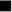) a growing crystal and (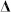) experiments with the empty platform^12^.

**Figure 6:**
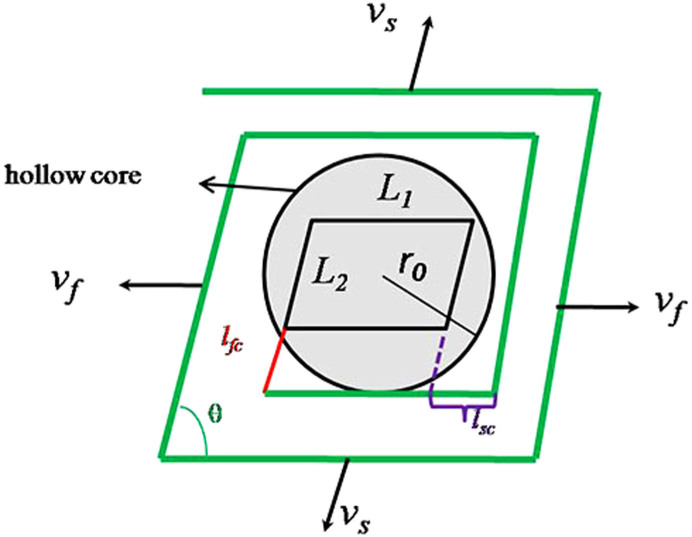
Model of a hillock with a core.

**Figure 7:**
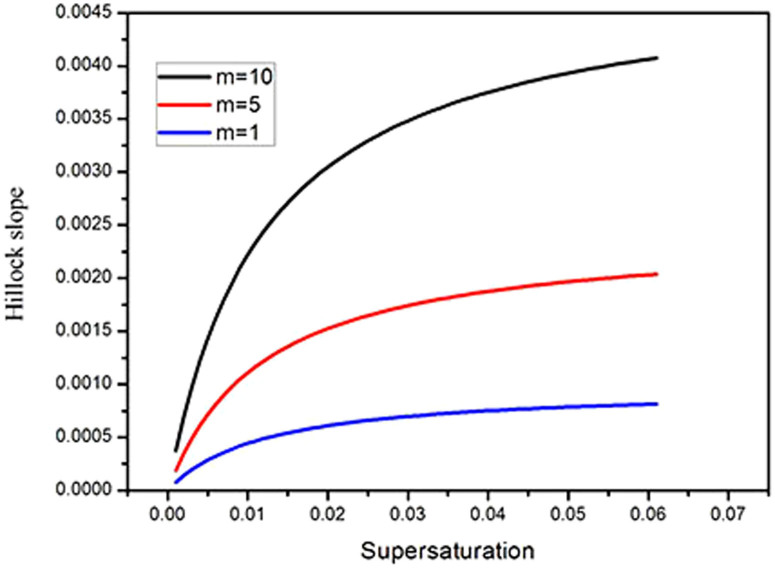
Hillock slope versus supersaturation along with curves those predicted by equation (7) for *L_1_* = 100 nm, *L_2_* = 86 nm at m = 1, m = 5, and m = 10.

The growth solutions of KDP were prepared by dissolving KDP salt (≥99.9% purity) in 18 MΩ de-ionized water. The solutions were filtered by using a polysulfone filter with a pore diameter of 0.1 μm. Crystallization was performed in a 5000 ml glass container. Holden-type crystallizers with temperature control accuracy of ±0.1°C were used throughout the experiments. All KDP crystallization solutions had initial growth temperatures of 56°C. The supersaturation was controlled by reducing the temperature of the growth solution, which could be calculated as: 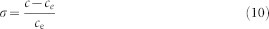


The KDP point seeds with a defined crystallographic direction of 59° with respect to the Z axis, which is shown in Figure 1, were used in our experiments. The crystal was rotated in a ‘forward-stop-backward’ mode with a speed of 77 rpm.

### Imaging experiments

Ex situ AFM measurements were performed using a Bruker Dimension Icon Scanning Probe Microscope (SPM) system in ScanAsyst mode with standard SiN cantilevers^23^. KDP crystal samples used for AFM characterization were grown from solutions with varying supersaturation values.

## Author Contributions

X.S. put forward this research direction. F.L., L.Z. and H.Z. contributed to crystal growth. G.Y. carried out the AFM experiments. F.L., X.G., H.R., L.Z., M.X., B.W., Q.G. and X.G. analyzed data from experiments. B.L., L.L. and S.J. revised this paper. H.Z. proposed advance for image processing. All authors disscussed this results.

## Figures and Tables

**Table 1:** Average value of hillock slops in different supersaturations

*σ*	0.007	0.01	0.015	0.018	0.02	0.04	0.055	0.06
P	0.00181	0.00302	0.00369	0.00439	0.00472	0.00501	0.00518	0.00537

**Table 2:** Values of parameters^6^ used in all evaluating equations

*α* (erg cm^−2^)	*ω* (*cm^3^* *mole^−1^*)	*h* (*cm*)	*E_as_* (ev molecule^−1^)	*E_af_* (ev molecule^−1^)	*β_0s_* (cm s^−1^)	*β_0f_* (cm s^−1^)
24	9.68 × 10^−23^	3.7 × 10^−8^	0.26	0.21	2 × 10^3^	6.54 × 10^2^
